# BRD7 facilitates ferroptosis via modulating clusterin promoter hypermethylation and suppressing AMPK signaling in diabetes-induced testicular damage

**DOI:** 10.1186/s10020-024-00868-x

**Published:** 2024-07-12

**Authors:** Yuehai Xiao, Zongjian Liang, Jun Qiao, Zhiqiang Zhu, Bei Liu, Yuan Tian

**Affiliations:** 1 Department of Urology, Affiliated Hospital of Guizhou Medical University, Clinical Medical College of Guizhou Medical University, No.28 Guiyi Street, Yunyan District, Guiyang, Guizhou Province 550004 China; 2 Department of Urology, School of Nursing, Affiliated Hospital of Guizhou Medical University, Guizhou Medical University, Guiyang, Guizhou Province 550004 China

**Keywords:** BRD7, Clusterin, Hypermethylation, AMPK, Diabetes-induced testicular damage

## Abstract

**Background:**

Diabetes mellitus (DM)-induced testicular damage is associated with sexual dysfunction and male infertility in DM patients. However, the pathogenesis of DM-induced testicular damage remains largely undefined.

**Methods:**

A streptozotocin (STZ)-induced diabetic model and high glucose (HG)-treated in vitro diabetic model were established. The histological changes of testes were assessed by H&E staining. Serum testosterone, iron, MDA and GSH levels were detected using commercial kits. Cell viability and lipid peroxidation was monitored by MTT assay and BODIPY 581/591 C11 staining, respectively. qRT-PCR, immunohistochemistry (IHC) or Western blotting were employed to detect the levels of BRD7, Clusterin, EZH2 and AMPK signaling molecules. The associations among BRD7, EZH2 and DNMT3a were detected by co-IP, and the transcriptional regulation of Clusterin was monitored by methylation-specific PCR (MSP) and ChIP assay.

**Results:**

Ferroptosis was associated with DM-induced testicular damage in STZ mice and HG-treated GC-1spg cells, and this was accompanied with the upregulation of BRD7. Knockdown of BRD7 suppressed HG-induced ferroptosis, as well as HG-induced Clusterin promoter methylation and HG-inactivated AMPK signaling in GC-1spg cells. Mechanistical studies revealed that BRD7 directly bound to EZH2 and regulated Clusterin promoter methylation via recruiting DNMT3a. Knockdown of Clusterin or inactivation of AMPK signaling reverses BRD7 silencing-suppressed ferroptosis in GC-1spg cells. In vivo findings showed that lack of BRD7 protected against diabetes-induced testicular damage and ferroptosis via increasing Clusterin expression and activating AMPK signaling.

**Conclusion:**

BRD7 suppressed Clusterin expression via modulating Clusterin promoter hypermethylation in an EZH2 dependent manner, thereby suppressing AMPK signaling to facilitate ferroptosis and induce diabetes-associated testicular damage.

**Supplementary Information:**

The online version contains supplementary material available at 10.1186/s10020-024-00868-x.

## Introduction

Diabetes mellitus (DM) is a metabolic disease with high incidence worldwide, and it is accompanied with long-term hyperglycemia due to insulin deficiency and resistance (Cole and Florez 2020). Sustained hyperglycemia leads to different diabetes-related complications, including male reproductive dysfunction (He et al. 2021a, b). As the number of young-onset DM (< 40-year-old) is increasing, male reproductive dysfunction and infertility have gained more attentions in recent years (Magliano et al. 2020; Standl et al. 2019). Accumulating evidence suggests that hyperglycemia-induced excess oxidative stress, abnormal reproductive hormone production, altered zinc metabolism and inflammation contribute to the onset and progression of DM-induced testicular damage (He et al. 2021a, b; Ding et al. 2015). Blood sugar control remains the key treatment of DM-induced testicular dysfunction (He et al. 2021a, b), however, the efficacies of most available antidiabetic agents on male reproduction remain largely undefined (Tavares et al. 2019). The effective therapeutic strategy for DM-induced male reproductive dysfunction is urgently needed. It is of great clinical significance to unravel the mechanism underlying DM-induced testicular dysfunction.

Ferroptosis, a novel form of programmed cell death, is characterized by iron-dependent accumulation of lipid peroxidation (Jiang et al. 2021). It is well-established that SLC7A11/GPX4 axis serves as one of the most critical antioxidant defenses against ferroptosis (Zheng and Conrad 2020). Emerging evidence supports the role of ferroptosis in male reproductive disorders (Yuan et al. 2023). For instance, a recent study has illustrated that Deoxynivalenol triggers testicular ferroptosis by suppressing Nrf2/SLC7A11/GPX4 axis (Yang et al. 2023). However, the role of ferroptosis in DM-induced testicular damage is still uninvestigated. Previous study has reported that Clusterin is downregulated in the testis from diabetic rat and high-glucose-treated GC-1spg cells (Tian et al. 2021). More importantly, loss of Clusterin causes renal lipid accumulation and lipid metabolism-associated kidney disease (Heo et al. 2018), raising the possibility that Clusterin might regulate DM-induced testicular damage by modulating ferroptosis. Mechanistic studies have demonstrated that Clusterin activates AMPK signaling which is known to inhibit ferroptosis (Lee et al. 2020; Park et al. 2020). In addition, Clusterin is also downregulated via promoter hypermethylation in rat fibroblasts and human cancer cells (Lund et al. 2006; Serrano et al. 2009). Whether the promoter hypermethylation of Clusterin and AMPK activation are implicated in the DM-induced testicular damage merits in-depth investigation.

A recent study has demonstrated that METTL3-mediated m^6^A modification enhances TUG1 stability and further increases Clusterin mRNA stability via recruiting SRSF1, thereby alleviating DM-induced testicular damage (Tian et al. 2023). In addition to post-transcriptional RNA modification, transcriptional silencing of target genes might also be implicated in the regulation of DM-induced testicular damage. Previous report has showed that bromodomain protein 7 (BRD7) interacts with polycomb repressive complex 2 (PRC2), in particular the core subunits SUZ12 and EZH2, to mediate transcriptional repression of target genes (Tae et al. 2011). EZH1 or EZH2 in PRC2 complexes catalyzes H3K27 methylation (Margueron and Reinberg 2011), suggesting that BRD7 might regulate downstream gene expression via EZH2-dependent H3K27 methylation. It is worth noting that BRD7 enhances ferroptosis in hepatic stellate cells (HSCs) (Zhang et al. 2020), and DM upregulates myocardial BRD7 expression (Wang et al. 2017). However, the biological role of BRD7 in DM-induced testicular damage is still elusive.

In summary, we aim to investigate the mechanism underlying DM-induced testicular damage. In this study, we demonstrated that ferroptosis was associated with DM-induced testicular damage in vivo and in vitro. BRD7 interacted with EZH2 to recruit DNMT3a, thereby regulating Clusterin promoter methylation and suppressing its expression. In vivo study further showed that lack of BRD7 ameliorated DM-induced testicular damage and ferroptosis via upregulating Clusterin expression and activating AMPK signaling. This study unraveled a novel regulatory mechanism of DM-induced testicular damage, and shed light on the targeted therapeutic strategies for DM-induced testicular damage.

## Materials and methods

### Animal study

Male C57BL/6J mice (5 ~ 6-week-old, *n* = 6 in each group) were from Hunan Slake Jingda Co., Ltd (Changsha, China). All animal studies were approved by Affiliated Hospital of Guizhou Medical University, Clinical Medical College of Guizhou Medical University. For the induction of diabetes, mice were injected with streptozotocin (STZ, 45 mg/kg per day in 0.1 M citric acid buffer, pH 4.5, S0130, Sigma-Aldrich, St. Louis, MO, USA) intraperitoneally for 5 days. Equal volume of vehicle control (0.1 M citric acid buffer, pH 4.5) was administrated into control mice intraperitoneally. For BRD7 knockdown study in diabetic mice, AAV2/2-U6-shBRD7 and corresponding control were obtained from GeneChem (Shanghai, China). AAV2/2-U6-shBRD7 (2.6 × 10^12^ genome copies (GC)/mL) was injected through tail vein 2 weeks prior to STZ administration as described (Zheng et al. 2019).

### Cell culture and treatment

Mouse germ cell line GC-1spg cells were purchased from ATCC (Manassas, VA, USA). GC-1spg cells were cultured in DMEM supplemented with 10% FBS (Thermo Fisher Scientific, Grand Island, NY, USA), and maintained at 37 °C with 5% CO_2_ in air. For high glucose (HG) treatment, GC-1spg cells were treated with 30 mM glucose for 24 h. GC-1spg cells were treated with ferroptosis inhibitor Ferrostatin-1 (2 µM, SML0583), caspase inhibitor Z-VAD-FMK (20 µM, V116), necroptosis inhibitor Necrostatin-1 (50 µM, N9037), DNMT inhibitor 5-Azacytidine (5 µM, A1287) or AMPK inhibitor Compound C (20 µM, 171,261) for 24 h. D-Mannitol (25 mM, M4125) was used as an osmotic stimulation (OS) control. All chemicals were purchased from Sigma-Aldrich.

### Cell transfection

ShNC, shBRD7#1, shBRD7#2, shEZH2 and shClusterin were from RiboBio (Guangzhou, China). The full-length of BRD7 was cloned into pcDNA3.1 (Invitrogen, Carlsbad, CA, USA). GC-1spg cells were transfected with shRNA or overexpression plasmid using Lipofectamine 2000 (11,668,019, Invitrogen). Cells were harvested at 48 h post-transfection for subsequent analysis.

### Hematoxylin and Eosin (H&E) staining

Mouse testes were fixed with Bouin’s solution (HT10132, Sigma-Aldrich) for 12 h and embedded in paraffin. The sections were stained with H&E solution (H3136 and HT110132, Sigma-Aldrich) as described (Ma et al. 2019). Images were acquired using a microscope (Nikon, Tokyo, Japan).

### Immunohistochemistry (IHC) and immunofluorescence (IF) staining

Frozen mouse testis tissues were sectioned and fixed with 4% paraformaldehyde (PFA), and permeabilized with 0.3% Triton X-100. The slides were blocked with 10% normal goat serum, followed by the incubation with anti-BRD7 (1:100, ab307140, Abcam, Cambridge, UK), antibody or normal rabbit IgG (10,500 C, Invitrogen) at 4 °C overnight. The section was then incubated with anti-rabbit secondary antibody (1:5000, 31,460, Invitrogen) for 1 h. Signals were visualized using DAB substrate (P0203, Beyotime, Haimen, China). For co-staining of BRD7 and SOX17, slides were incubated with anti-BRD7 (1:100, ab252820) and anti-SOX17 (1:100, ab224637) antibodies at 4 °C overnight. This was followed by the incubation with Alexa Fluor 488-conjugated anti-rabbit or Alexa Fluor 555-conjugated anti-rat secondary antibodies (dilution, vendor). Images were acquired by confocal microscope (Nikon).

### ELISA assay

Whole mouse blood samples were collected and allowed to clot for 30 min. After centrifugation at 1000 g for 10 min, serum (supernatant) was collected. The serum level of testosterone was assessed using Mouse/Rat Testosterone ELISA kit (JL25196, Jonln, Shanghai). A450 was measured using a microplate reader (Thermo Fisher Scientific, Waltham, MA, USA).

### Measurement of iron, MDA and GSH

The iron level in the testis or GC-1spg cells was determined using Iron Assay Kit (ab83366, Abcam). Briefly, tissues or cells were homogenized in Iron Assay Buffer. After centrifugation, iron standard or samples were then incubated with Iron Probe at room temperature for 1 h. A593 was measured using a microplate reader. The level of MDA in testis or cells was measured using MDA Assay Kit (JL53632, Jonln, Shanghai). In brief, MDA standard or samples were incubated with MDA Color Reagent at room temperature for 30 min, followed by the incubation with Reaction Solution at room temperature for 1 h. A450 was measured using a microplate reader. GSH level was determined using GSH Assay kit (ab239727, Abcam). Briefly, tissue or cell lysates were prepared with 5% sulfosalicylic acid. Diluted samples and standards (20 µL) were incubated with Reaction Mix (80 µL) for 60 min. A450 was measured using a microplate reader (Zhou et al. 2022).

### Measurement of lipid peroxidation

Lipid peroxidation was detected using BODIPY 581/591 C11 (D3861, Invitrogen). Cells were stained the 10 µM BODIPY 581/591 C11 at 37 °C for 30 min. Cells were then trypsinized and analyzed by flow cytometry at 488 nm excitation with 530/30 BP filter (BD Biosciences, San Jose, CA, USA).

### MTT assay

Cell viability was monitored using MTT assay kit (M8180, Solarbio, Beijing, China) as described. Cells were plated into 96-well plates 24 h prior to different treatments. At the designated time-points, cells were incubated with MTT solution (10 µL per well) for 4 h, and Formazan (110 µL per well) was added into each well. A490 was measured using a microplate reader (Thermo Fisher Scientific).

### RNA isolation and qRT-PCR

Total RNA from testis tissue or GC-1spg cells was extracted using Trizol (15596026, Invitrogen). cDNAs were synthesized using SuperScript III reverse transcriptase (18080093, Invitrogen). qRT-PCR was conducted using SYBR Premix Ex Taq (RR039W, TaKaRa, Dalian, China). The expression of target gene was calculated using 2^–ΔΔCT^ method. GAPDH was used as internal control. The following primers were used in this study: BRD7 F: 5’-TCAGGAGGCAAGCTAACACG-3’, R: 5’-AATGCTCTGGTCTGGCCTTC-3’; EZH2 F: 5’-AGGACGGCTCCTCTAACCAT-3’, R: 5’-AGCCAGGTAGCATGGACACT-3’; Clusterin F: 5’-CGAAGATGCTCAACACCTCA-3’, R: 5’- TGTGATGGGGTCAGAGTCAA-3’ and GAPDH F: 5’- AGCCCAAGATGCCCTTCAGT-3’, R: 5’- CCGTGTTCCTACCCCCAATG-3’.

### Methylation-specific PCR (MSP)

The methylation of the Clusterin promoter was determined by MSP using specific primers. PCR reaction was performed using bisulfite-treated DNA as a template, and PCR products were detected by 2% agarose gel electrophoresis.

### Western blot analysis

Protein lysates were prepare using RIPA lysis buffer (89,900, Pierce, Rockford, IL, USA). Equal amount of protein was separated by gel electrophoresis and transferred onto PVDF membrane (Pierce). After blocking, the blots were incubated with primary antibody at 4 °C overnight, and this was followed by the incubation with HRP-conjugated secondary antibody. Signals were detected using ECL Pico PLUS substrate (34,580, Pierce). Primary antibodies used in Western blotting: anti-SLC7A11 (1:1000, ab307601, Abcam), anti-GPX4 (1:1000, ab125066, Abcam), anti-BRD7 (1:1000, ab307140, Abcam), anti-Clusterin (1:500, PA5-46931, Invitrogen), anti-DNMT3a (1:500, ab307503, Abcam), anti-EZH2 (1:1000, ab307646, Abcam), anti-p-AMPKα (Thr172, 1:1000, #2535, Cell Signaling Technology, Danvers, MA, USA), anti-AMPK (1:1000, #2532, CST), anti-Nrf2 (1:1000, PA5-27882, Invitrogen) and anti-GAPDH (1:2000, ab8245, Abcam) antibodies.

### Co-immunoprecipitation (co-IP)

Cell lysates were prepared using IP lysis buffer. 1 mg cell lysates were incubated with anti-BRD7 (1:30, ab307140, Abcam), anti-DNMT3a antibody (1:30, ab307503, Abcam) or normal IgG at 4 °C overnight. The immunocomplexes were enriched by Protein A/G magnetic beads (88,802, Pierce). After rinsing, the elutes were analyzed by Western blotting. Whole cell lysates and normal IgG were used as an input control and negative control, respectively.

### Chromatin immunoprecipitation (ChIP) assay

ChIP assay was conducted using Pierce magnetic ChIP kit (26,157, Pierce). Briefly, cells were crosslinked with 1% formaldehyde, and the chromatin fragments were prepared by lysis and MNase digestion. The chromatin fragments were then incubated with anti-EZH2 (5 µg, ab307646, Abcam), anti-H3K27me3 (5 µg, ab6002, Abcam), anti-DNMT3a (5 µg, PA1882, Invitrogen) antibody or normal IgG at 4 °C overnight. The immunoprecipitated DNA was purified and analyzed by qRT-PCR. Total chromatins and normal IgG were employed as an input control and negative control, respectively.

### Statistical analysis

Data were presented as mean ± SD from at least 3 independent experiments. Statistical analyses were conducted using GraphPad Prism 8.0. The two-group and multi-group differences were analyzed by Student’s *t* test or one-way ANOVA. respectively. *P* < 0.05 was statistically significant.

## Results

### Ferroptosis is associated with testicular damage in diabetic mice and HG-treated GC-1spg cells

**Fig. 1:**
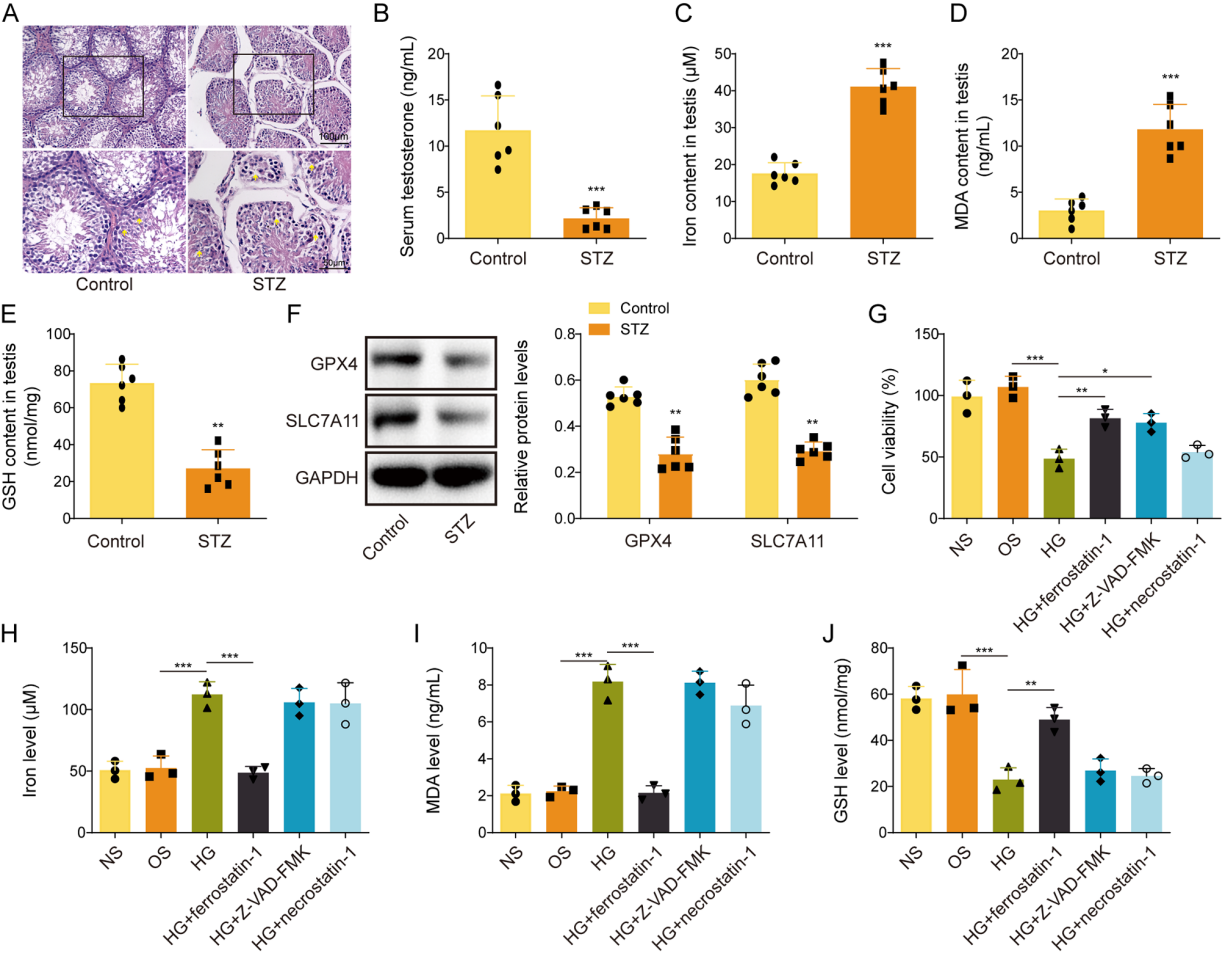
Ferroptosis is associated with testicular damage in diabetic mice and high glucose-treated GC-1spg cells. **(A)** The histological changes of mouse testis were assessed by H&E staining. **(B)** The mouse serum testosterone level was measured using ELISA kit. **(C-E)** The levels of iron, MDA and GSH in mouse testis were detected using commercial kits. **(F)** The protein levels of GPX4 and SLC7A11 were detected by Western blotting. **(G)** Cell viability of GC-1spg cells was monitored by MTT assay. **(H-J)** The levels of iron, MDA and GSH in GC-1spg cells were detected using commercial kits. *, *P* < 0.05; **, *P* < 0.01; ***, *P* < 0.001. NG, normal glucose; OS, osmotic stimulation; HG, high glucose

We first examined the histological changes of testis in STZ-induced diabetic mouse model. H&E staining showed that intact seminiferous tubules structures and Leydig cells were observed in control mice, whereas the seminiferous tubules in STZ mice were disrupted with significant loss of spermatogenic cells and vacuolization (Fig. 1A). The serum testosterone level was remarkably decreased in STZ mice, in comparison with that of control mice (Fig. 1B). In addition, the testis tissues derived from STZ mice exhibited increased iron and MDA levels, along with decreased GSH level (Fig. 1C-E). This was accompanied with the downregulation of GPX4 and SLC7A11 in the testis from STZ mice (Fig. 1F). MTT assay further revealed that HG-impaired cell viability of GC-1spg cells was rescued by ferroptosis inhibitor Ferrostatin-1 or caspase inhibitor Z-VAD-FMK, but not by necroptosis inhibitor Necrostatin-1 (Fig. 1G). In GC-1spg cells, HG induced the levels of iron and MDA, but reduced GSH levels (Fig. 1H-J). Intriguingly, Ferrostatin-1 reversed HG-mediated changes of iron, MDA and GSH, while Z-VAD-FMK or Necrostatin-1 showed no rescue effect on iron, MDA and GSH levels in GC-1spg cells (Fig. 1H-J). Collectively, these data indicate that ferroptosis is associated with testicular damage in diabetic mice, and ferroptosis is also triggered by HG in GC-1spg cells.

### BRD7 is highly expressed in the testis from STZ mice and HG-treated GC-1spg cells

**Fig. 2:**
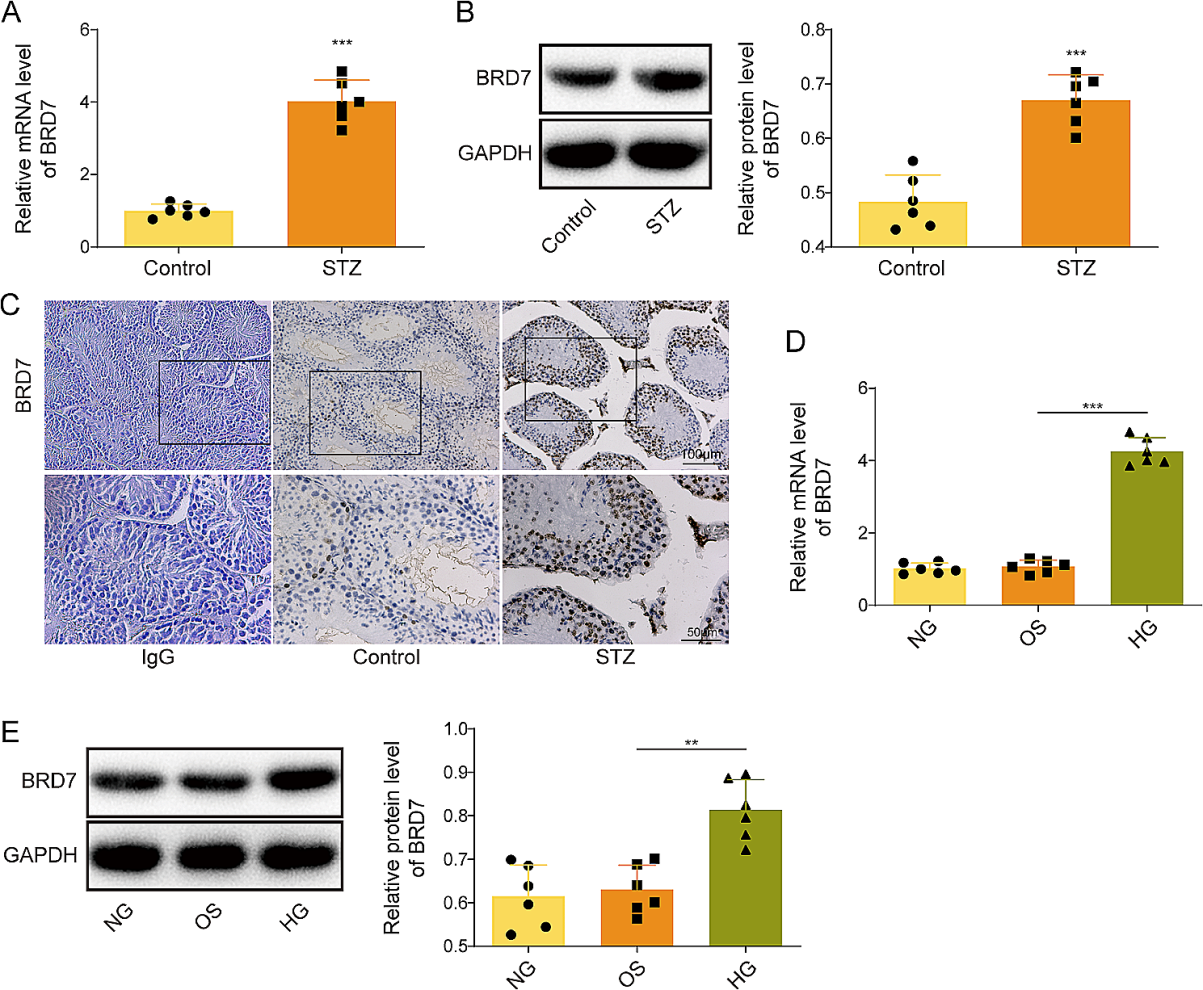
BRD7 is highly expressed in the testis from STZ mice and HG-treated GC-1spg cells. **(A)** The mRNA level of BRD7 in mouse testis was detected by qRT-PCR. **(B)** The protein level of BRD7 in mouse testis was detected by Western blotting. **(C)** The expression level of BRD7 in mouse testis was detected by IHC analysis. Normal rabbit IgG was used as a negative control. GC-1spg cells were treated with NG, OS or HG. **(D)** The RNA level of BRD7 in GC-1spg cells was detected by qRT-PCR. **(E)** The protein level of BRD7 in GC-1spg cells was detected by Western blotting. *, *P* < 0.05; ***, *P* < 0.001. NG, normal glucose; OS, osmotic stimulation; HG, high glucose

To delineate the biological role of BRD7 in diabetes-induced testicular damage, the expression of BRD7 was examined in vivo and in vitro. qRT-PCR and Western blotting showed that BRD7 was upregulated in the testis tissues derived from STZ mice (Fig. 2A&B). Consistently, increased immunoreactivity of BRD7 was also observed in the seminiferous tubules of STZ mice, compared with that of control mice as detected by IHC analysis (Fig. 2C). In IgG group, no signal was detected in the seminiferous tubules, indicating the specificity of BRD7 immunoreactivity (Fig. 2C). In accordance with the in vivo findings, HG dramatically induced BRD7 expression, compared with NG or OS control group (Fig. 2D&E). These findings suggest that BRD7 is elevated in the testis from STZ mice and HG-treated GC-1spg cells, and it may play a critical role in diabetes-induced testicular damage.

### Knockdown of BRD7 suppresses HG-induced ferroptosis in GC-1spg cells

**Fig. 3:**
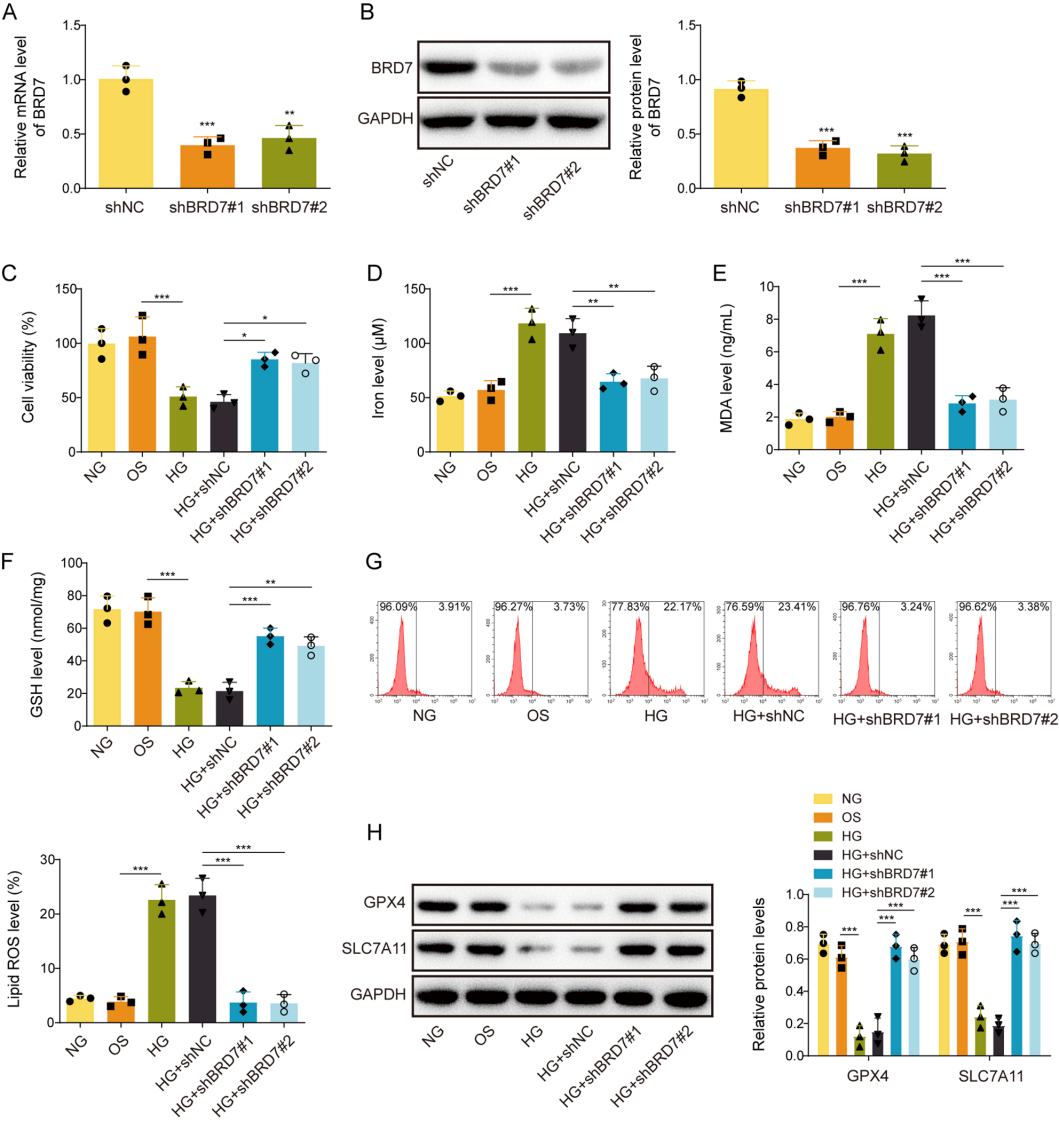
Knockdown of BRD7 suppresses HG-induced ferroptosis in GC-1spg cells. GC-1spg cells were transfected with shBRD7 or negative control shRNA (shNC), followed by the treatment of HG. **(A)** The mRNA level of BRD7 in GC-1spg cells was detected by qRT-PCR. **(B)** The protein level of BRD7 in GC-1spg cells was detected by Western blotting. **(C)** Cell viability of GC-1spg cells was monitored by MTT assay. **(D-F)** The levels of iron, MDA and GSH in GC-1spg cells were detected using commercial kits. **(G)** Lipid ROS in GC-1spg cells was monitored by BODIPY 581/591 C11 staining with flow cytometry analysis. **(H)** The protein levels of GPX4 and SLC7A11 in GC-1spg cells were detected by Western blotting. *, *P* < 0.05; **, *P* < 0.01; ***, *P* < 0.001. NG, normal glucose; OS, osmotic stimulation; HG, high glucose

Loss-of-function study was next conducted to investigate the effect of BRD7 on ferroptosis in GC-1spg cells. As anticipated, transfection of shBRD7#1 or shBRD7#2 successfully downregulated BRD7 mRNA and protein levels in GC-1spg cells (Fig. 3A&B). MTT assay showed that silencing of BRD7 protected against HG-impaired cell viability in GC-1spg cells (Fig. 3C). Moreover, HG-mediated changes of iron, MDA and GSH levels were also reversed by BRD7 knockdown (Fig. 3D-F). Flow cytometry further revealed that lack of BRD7 counteracted HG-induced lipid ROS in GC-1spg cells (Fig. 3G). HG-downregulated GPX4 and SLC7A11 in GC-1spg cells were also attenuated by BRD7 knockdown (Fig. 3H). These findings indicate that BRD7 is implicated in the regulation of ferroptosis in GC-1spg cells.

### Knockdown of BRD7 inhibits HG-induced Clusterin promoter methylation and HG-inactivated AMPK signaling in GC-1spg cells

**Fig. 4:**
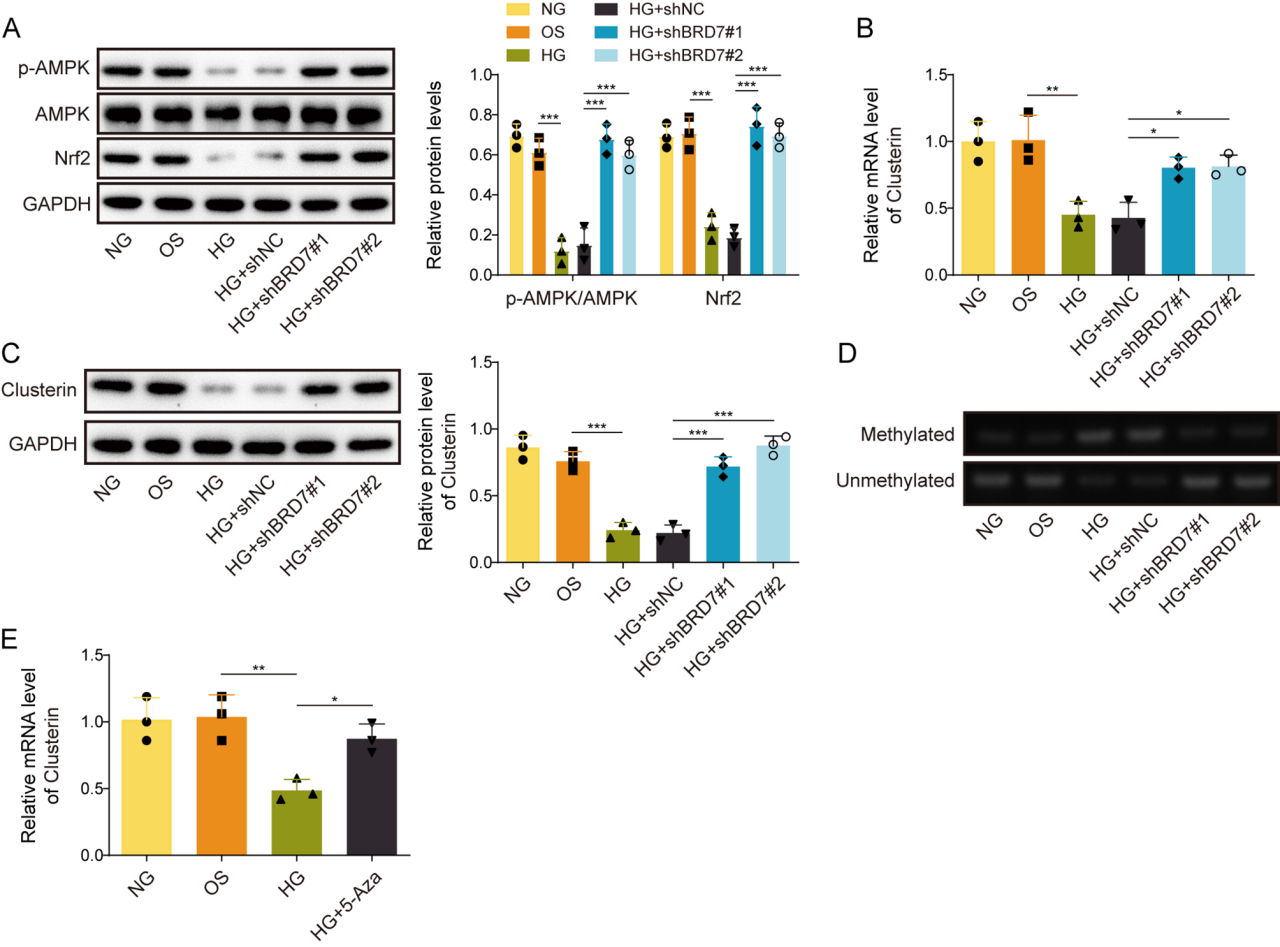
Knockdown of BRD7 inhibits HG-induced Clusterin promoter methylation and AMPK signaling in GC-1spg cells. GC-1spg cells were transfected with shBRD7 or shNC, followed by the treatment of HG. **(A)** The protein levels of p-AMPK, AMPK and Nrf2 in GC-1spg cells were detected by western blot. **(B)** The mRNA level of Clusterin in GC-1spg cells was detected by qRT-PCR. **(C)** The protein level of Clusterin in GC-1spg cells was detected by Western blotting. **(D)** The Clusterin promoter methylation was monitored by MSP. GC-1spg cells were treated with HG or/and 5-Azacytidine. **(E)** The mRNA level of Clusterin in GC-1spg cells was detected by qRT-PCR. *, *P* < 0.05; **, *P* < 0.01; ***, *P* < 0.001. 5-Aza, 5-Azacytidine; NG, normal glucose; OS, osmotic stimulation; HG, high glucose

We next sought to study the mechanism underlying BRD7-regulated ferroptosis in GC-1spg cells. Western blotting revealed that HG inactivated AMPK and Nrf2 in GC-1spg cells in which the protein levels of p-AMPK and Nrf2 were downregulated by HG (Fig. 4A). Interestingly, silencing of BRD7 led to rebounds of p-AMPK and Nrf2 in the presence of HG (Fig. 4A). Previous study has illustrated that Clusterin exhibited protective effects in NAFLD and western diet-induced obesity through activating AMPK and Nrf2 (Park et al. 2020). We thus detected the expression of Clusterin in GC-1spg cells by qRT-PCR and Western blotting. As presented in Fig. 4B&C, BRD7 knockdown reversed HG-decreased Clusterin expression in GC-1spg cells. MSP further showed that HG-induced Clusterin promoter methylation was also abrogated by BRD7 knockdown (Fig. 4D). Additionally, 5-Azacytidine, a DNA methyltransferase (DNMT) inhibitor, counteracted HG-reduced Clusterin mRNA level (Fig. 4E), suggesting that HG downregulated Clusterin via modulating DNA methylation. Together, these data indicate that silencing of BRD7 inhibits HG-induced Clusterin promoter methylation and HG-suppressed AMPK signaling in GC-1spg cells.

### BRD7 regulates Clusterin promoter methylation via recruiting DNMT3a in an EZH2-dependent manner

**Fig. 5:**
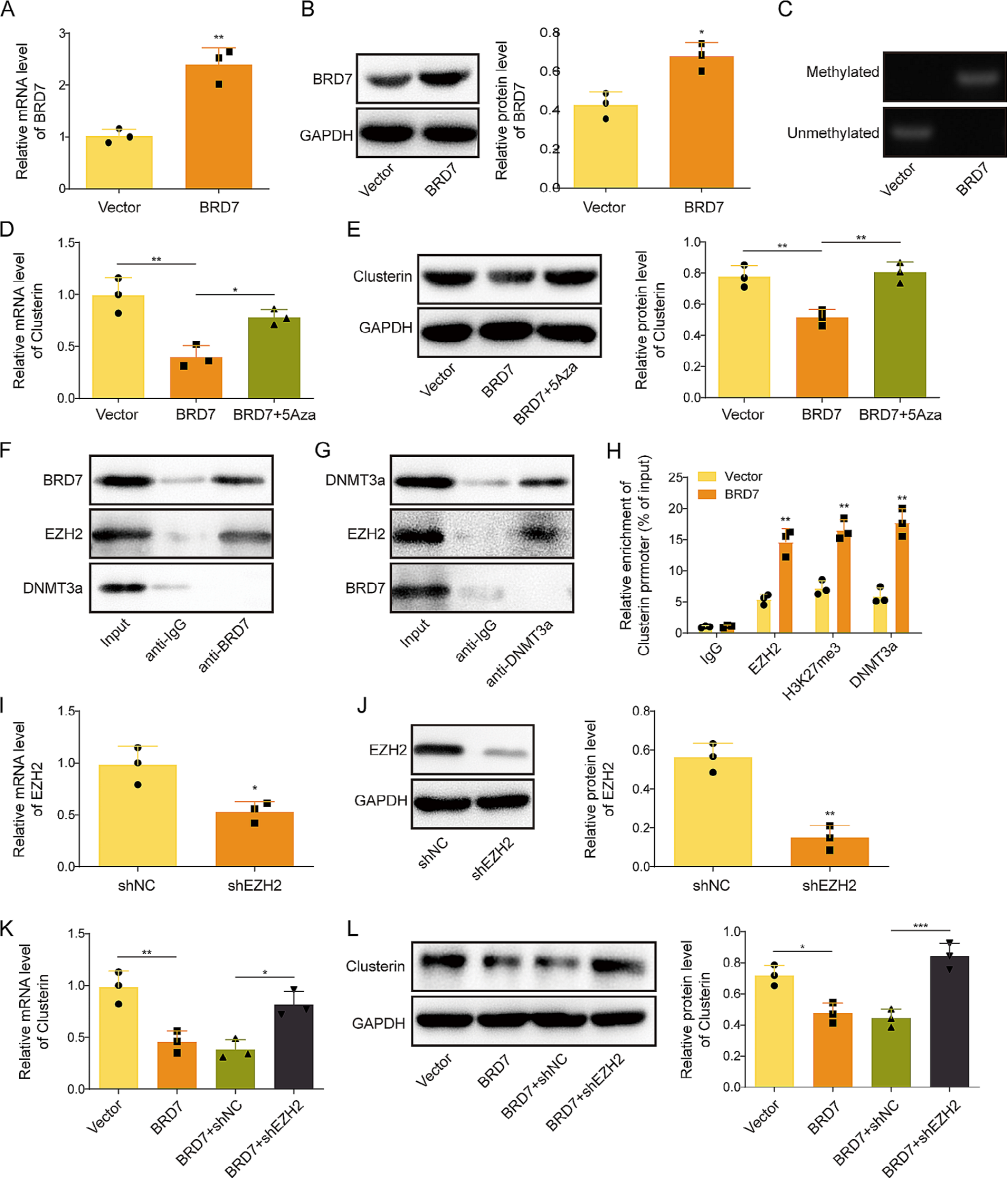
BRD7 regulates Clusterin promoter methylation via recruiting DNMT3a in an EZH2-dependent manner. GC-1spg cells were transfected with BRD7 overexpression plasmid or vector alone. (**A**&**B**) The mRNA and protein levels of BRD7 in GC-1spg cells were detected by qRT-PCR and Western blotting, respectively. **(C)** The Clusterin promoter methylation was monitored by MSP. GC-1spg cells were transfected with BRD7 overexpression plasmid or vector alone, followed by the treatment with 5-Azacytidine. (**D**&**F**) The mRNA and protein levels of Clusterin in GC-1spg cells were detected by qRT-PCR and Western blotting, respectively. (**F**&**G**) The interactions among BRD7, EZH2 and DNMT3a were detected by co-IP. Whole cell lysates or normal IgG was used as an input or negative control, respectively. **(H)** The interactions among EZH2, H3K27me3, DNMT3a and Clusterin promoter were assessed by ChIP assay. Total chromatin or normal IgG was used as an input or negative control, respectively. GC-1spg cells were transfected with shEZH2 or shNC. (**I**&**J**) The mRNA and protein levels of EZH2 in GC-1spg cells were detected by qRT-PCR and Western blotting, respectively. GC-1spg cells were transfected with BRD7 overexpression plasmid or/and shEZH2. (**K**&**L**) The mRNA and protein levels of Clusterin in GC-1spg cells were detected by qRT-PCR and Western blotting, respectively. *, *P* < 0.05; **, *P* < 0.01; ***, *P* < 0.001. 5-Aza, 5-Azacytidine

Previous study has demonstrated that BRD7 regulates downstream gene expression via PRC2-mediated epigenetic regulation (Tae et al. 2011). To further investigate the underlying mechanism by which BRD7 regulated Clusterin promoter methylation, overexpression study was conducted in GC-1spg cells. As shown in Fig. 5A&B, transfection of BRD7 overexpression construct successfully induced BRD7 mRNA and protein levels in GC-1spg cells. MSP revealed that reinforced BRD7 enhanced Clusterin promoter methylation (Fig. 5C). In addition, BRD7-suppressed Clusterin expression was attenuated by 5-Azacytidine (Fig. 5D&E), indicating that BRD7 regulated Clusterin expression through modulating DNA methylation. Interestingly, direct associations between BRD7 and EZH2, as well as between DNMT3a and EZH2, were detected by co-IP in GC-1spg cells (Fig. 5F&G). It is worth noting that no interaction was observed between BRD7 and DNMT3a (Fig. 5F&G). Moreover, ChIP assay revealed that antibodies against EZH2, H3K27me3 or DNMT3a successfully enriched Clusterin promoter, and these enrichments were increased in BRD7-overexpressing GC-1spg cells (Fig. 5H), suggesting that EZH2, H3K27me3 and DNMT3a are key players in BRD7-mediated regulation of Clusterin. Knockdown study further showed that shEZH2 reduced EZH2 expression in GC-1spg cells (Fig. 5I&J). BRD7-downregulated Clusterin was reversed by EZH2 knockdown as detected by qRT-PCR and Western blotting (Fig. 5K&L), indicating the indispensable role of EZH2 in BRD7-mediated regulation of Clusterin. These findings suggest that BRD7 interacts with EZH2 to recruit DNMT3a, thereby regulating Clusterin promoter methylation and its expression in GC-1spg cells.

### Knockdown of Clusterin or inactivation of AMPK signaling reverses BRD7 silencing-suppressed ferroptosis in GC-1spg cells

**Fig. 6:**
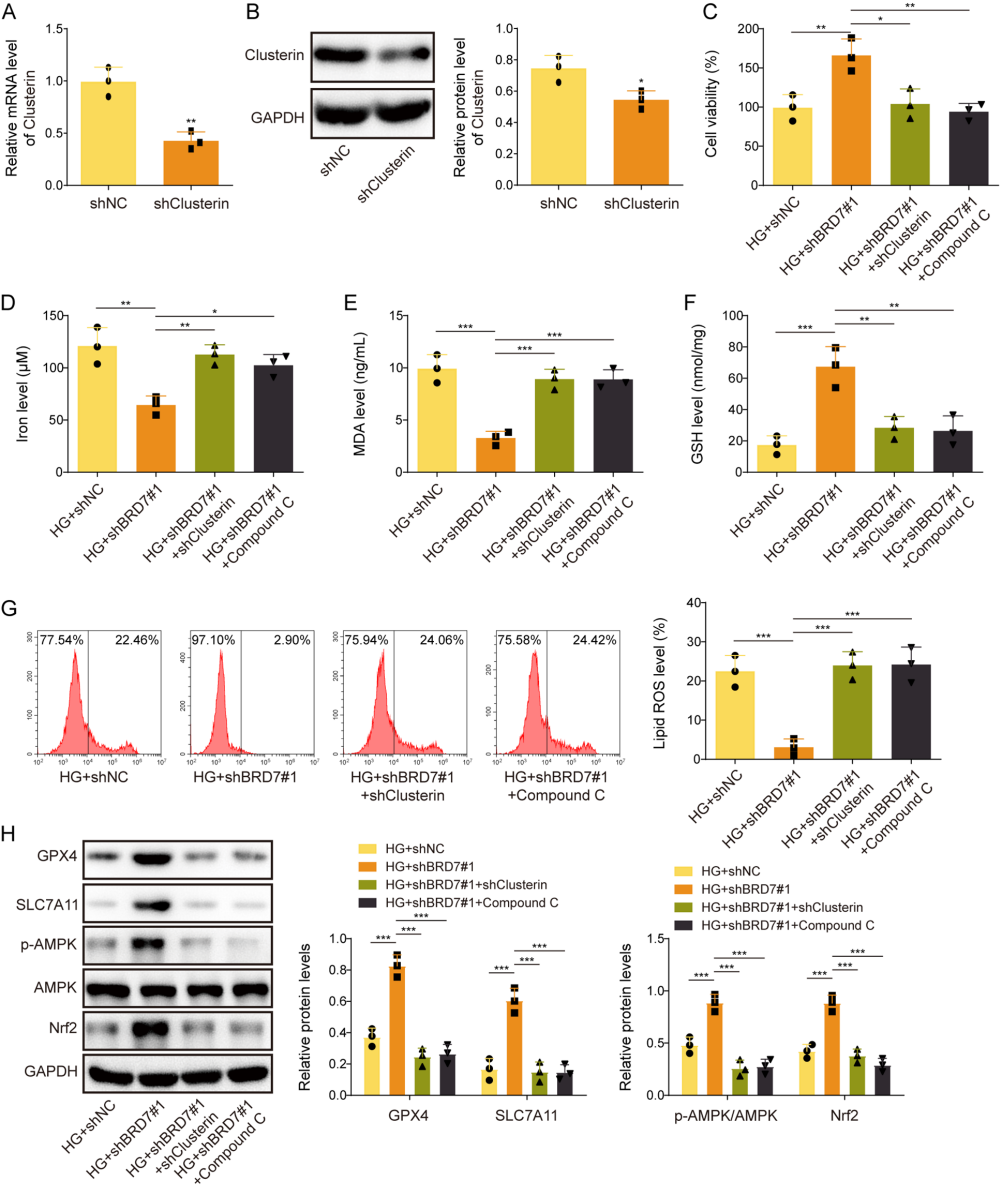
Knockdown of Clusterin or inactivation of AMPK signaling reverses BRD7 silencing-suppressed ferroptosis in GC-1spg cells. GC-1spg cells were transfected with shClusterin or shNC. (**A**-**B**) The mRNA and protein levels of Clusterin in GC-1spg cells were detected by qRT-PCR and Western blotting, respectively. GC-1spg cells were transfected with shBRD7#1 or/and shClusterin, followed by the treatment of HG or/and Compound C. **(C)** Cell viability of GC-1spg cells was monitored by MTT assay. (**D**-**F**) The levels of iron, MDA and GSH in GC-1spg cells were detected using commercial kits. **G** Lipid ROS in GC-1spg cells was monitored by BODIPY 581/591 C11 staining with flow cytometry analysis. (H) The protein levels of GPX4, SLC7A11, p-AMPK, AMPK and Nrf2 in GC-1spg cells were detected by Western blotting. *, *P* < 0.05; **, *P* < 0.01; ***, *P* < 0.001. HG, high glucose

To study the function of Clusterin in BRD7-regulated ferroptosis, loss-of-function experiments were carried out in GC-1spg cells. As anticipated, transfection of shClusterin remarkably decreased Clusterin expression in GC-1spg cells (Fig. 6A&B). MTT assay showed that the protective effect of shBRD7#1 on cell viability was abrogated by shClusterin or the AMPK inhibitor Compound C in HG-treated GC-1spg cells (Fig. 6C). Similarly, the rescue effects of shBRD7#1 on iron, MDA and GSH levels were also attenuated by shClusterin or Compound C in HG-treated GC-1spg cells (Fig. 6D-F). Consistent with these findings, flow cytometry showed that silencing of BRD7 protected against HG-induced lipid ROS, while this effect was counteracted by shClusterin or Compound C (Fig. 6G). In addition, BRD7 knockdown upregulated the protein levels of GPX4, SLC7A11, p-AMPK and Nrf2, whereas these changes were abolished by shClusterin or Compound C in HG-treated GC-1spg cells (Fig. 6H). These data suggest that silencing BRD7 suppresses HG-induced ferroptosis in GC-1spg cells via modulating Clusterin or/and AMPK signaling.

### Knockdown of BRD7 protects against diabetes-induced testicular damage and ferroptosis via increasing Clusterin to activate AMPK signaling in mice

**Fig. 7:**
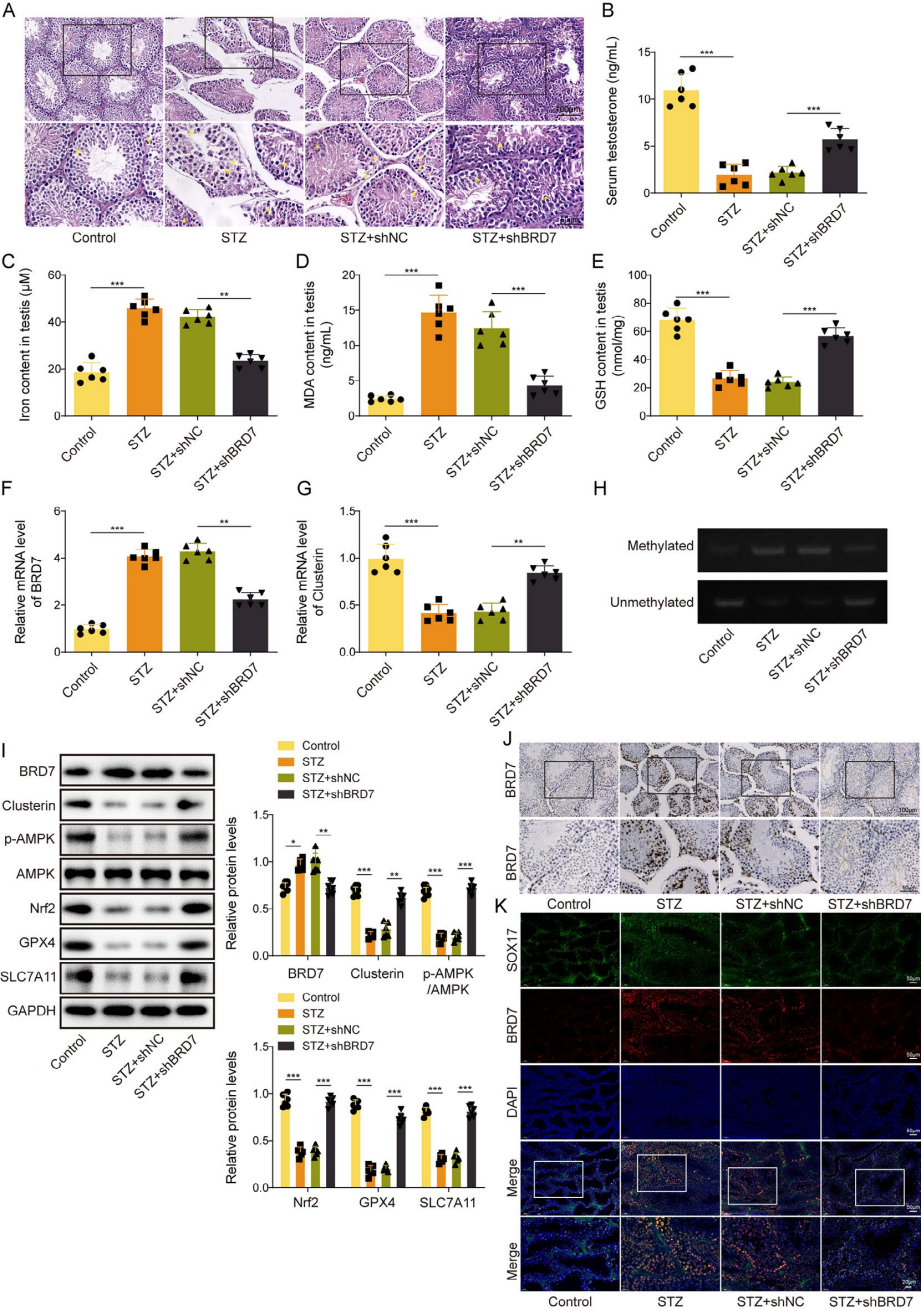
Knockdown of BRD7 protects against diabetes-induced testicular damage and ferroptosis via increasing Clusterin expression and activating AMPK signaling. **(A)** The histological changes of mouse testis were assessed by H&E staining. **(B)** The serum testosterone level in mice was measured using ELISA kit. (**C**-**E**) The levels of iron, MDA and GSH in mouse testis were detected using commercial kits. (**F**&**G**) The mRNA levels of BRD7 and Clusterin in the testis were detected by qRT-PCR. **(H)** The Clusterin promoter methylation in mouse testis was monitored by MSP. **(I)** The protein levels of BRD7, Clusterin, p-AMPK, AMPK, Nrf2, GPX4 and SLC7A11 were detected by Western blotting. **(J)** The immunoreactivities of BRD7 in the testis were detected by IHC analysis. **(K)** The immunoreactivities of BRD7 and SOX17 were assessed by IF staining. Blue, DAPI; Green, SOX17; Red, BRD7. *, *P* < 0.05; **, *P* < 0.01; ***, *P* < 0.001

The biological functions of BRD7 were next validated in STZ mice. As presented in Fig. 7A, H&E staining showed that lack of BRD7 alleviated STZ-disrupted seminiferous tubules in which the significant loss of spermatogenic cells and vacuolization were ameliorated by BRD7 knockdown. Additionally, STZ-decreased serum testosterone level was also rescued by shBRD7 as detected by ELISA assay (Fig. 7B). Consistently, STZ-induced changes of iron, MDA and GSH levels in the testis were also reversed by BRD7 knockdown (Fig. 7C-E). qRT-PCR, Western blotting and IHC analysis further showed that STZ-induced BRD7 expression was attenuated by shBRD7 in vivo (Fig. 7F, I, amp and J), and an opposite trend of Clusterin expression was observed in the testis (Fig. 7G and I). MSP showed that Clusterin promoter methylation was markedly increased in the testis of STZ mice, while knockdown of BRD7 attenuated this effect (Fig. 7H). Similar with Clusterin, STZ-decreased p-AMPK, Nrf2, GPX4 and SLC7A11 were counteracted by BRD7 knockdown in the testis (Fig. 7I). As shown in Fig. 7K, co-localization of BRD7 and SOX17 was elevated in the testis of STZ mice, whereas in vivo knockdown of BRD7 decreased co-localization of BRD7 and SOX17 in mouse testis tissues (Fig. 7K). These findings suggest that lack of BRD7 exhibits protective effects on diabetes-induced testicular damage and ferroptosis, possibly via inducing Clusterin expression and activating AMPK signaling.

## Discussion

**Fig. 8:**
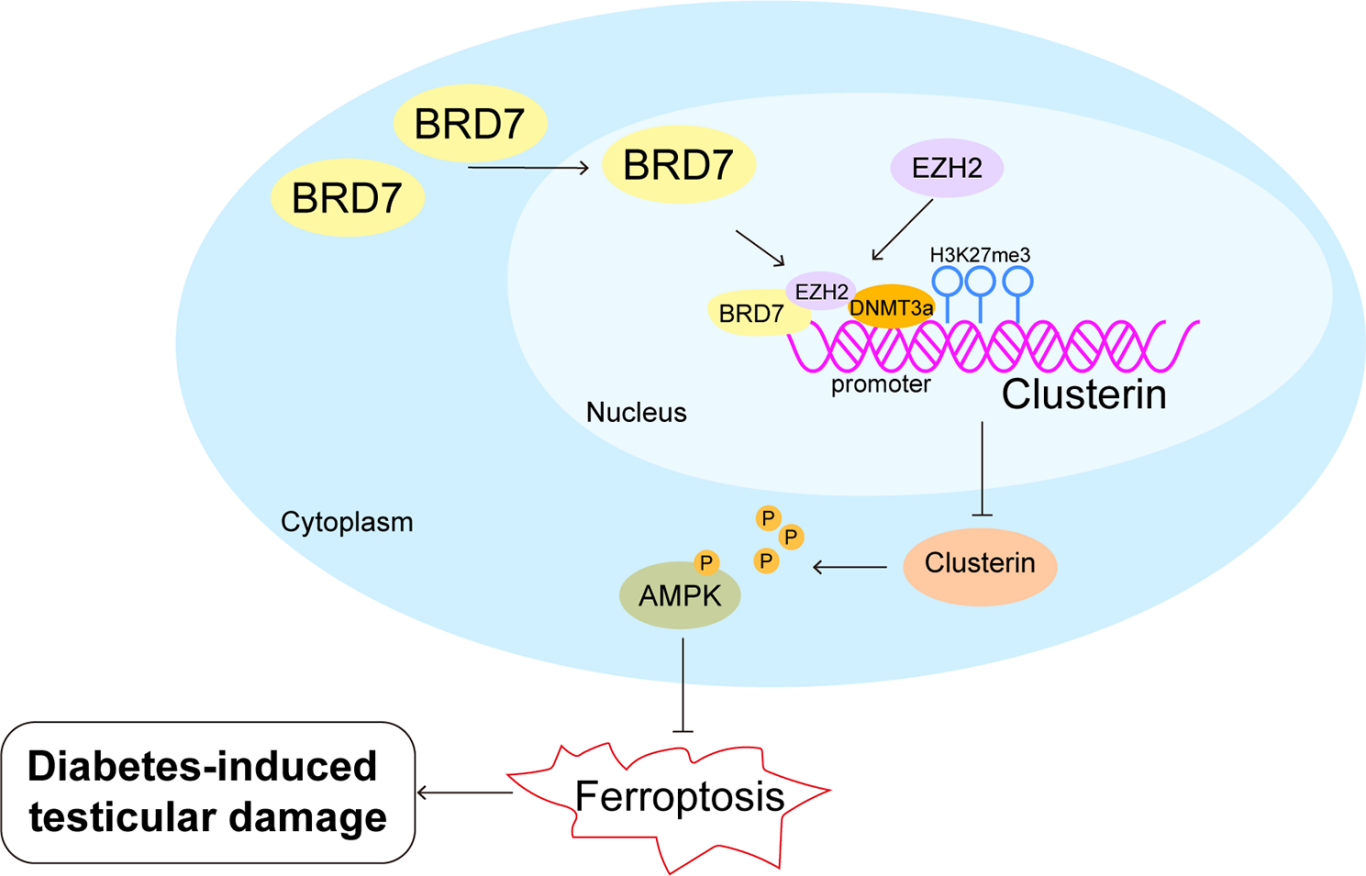
Graphic abstract of this study. BRD7 modulated Clusterin promoter hypermethylation in an EZH2/DNMT3a-dependent manner to suppress Clusterin expression, thus inactivating AMPK signaling to promote ferroptosis and induce DM-associated testicular damage

Approximately 90% diabetic men experience the decline in male fertility due to dysregulated testosterone level, reduction in sperm quality and semen volume (Ghazi et al. 2012; Maresch et al. 2018). Mechanistic studies have illustrated impaired structures of epididymides and testes in STZ-induced mouse or rat diabetic model (Long et al. 2018; Sampannang et al. 2018). These impairments could be attributed to engender hypogonadism, apoptosis and autophagy (He et al. 2021a, b). Our findings reported BRD7 modulated Clusterin promoter hypermethylation in an EZH2/DNMT3a-dependent manner to suppress Clusterin expression, thus inactivating AMPK signaling to promote ferroptosis and induce DM-associated testicular damage (Fig. 8). These findings broadened the understanding of DM-induced testicular damage and indicated that BRD7-associated ferroptosis has emerge as a therapeutic target for DM-induced male reproductive dysfunction.

BRD7 was originally identified as a novel bromodomain protein with ubiquitous expression in multiple tissues (Cuppen et al. 1999). Knockout study has illustrated the indispensable role of BRD7 in spermatogenesis in which depletion of BRD7 leads to arrest of spermatogenesis and male infertility in mice (Wang et al. 2016). A recent study has reported that *BRD7*-linked variants exhibit limited contribution to the impairment of spermatogenesis and male infertility in humans (He et al. 2021a, b). In the current study, we found that BRD7 was abnormally upregulated in STZ mouse testis which was associated with defective spermatogenesis, indicating that dysregulated BRD7, including up- or down-regulation, impairs spermatogenesis. In vitro study further confirmed that HG-induced BRD7 protein level was brought back to normal level by shBRD7, thus protecting against HG-induced ferroptosis in GC-1psg cells. This finding was similar with a recent report which demonstrated that knockout of BRD7 suppresses HSC ferroptosis (Zhang et al. 2020), suggesting that BRD7 triggers ferroptosis in different cells. Taken together, these data indicate that physiological BRD7 level is crucial for maintaining spermatogenesis.

A number of studies have demonstrated the role of BRD7 in different diseases via regulating gene transcription, chromatin remodeling, inflammation, unfolded protein response and cell cycle progression (Golick et al. 2018; Park and Lee 2020). However, the regulatory mechanism of BRD7 in DM-induced testicular damage remains ambiguous. As a subunit of polybromo-associated BRG1-associated factor (PBAF), BRD7 regulates gene transcription through binding to acetylated histone H3 and H4 in chromatin (Kaeser et al. 2008). BRD7 is involved in the regulation of cell cycle genes via binding to acetylated histones (Kzhyshkowska et al. 2003; Peng et al. 2006). In patient-derived B cells, BRD7 recruits PRMT5 and PRC2 to catalyze hypermethylation on histone H3R8, H4R3 and H3K27, thus regulating ST7 and RBL2 expression. This study has showed the direct interaction between BRD7 and EZH2 by GST pull-down assay (Tae et al. 2011), and this association was verified in GC-1spg cells by co-IP in this study. It is well-accepted that EZH2 catalyze H3K27me3 through interacting with DNMT1, DNMT3a and DNMT3b in cancer cells (Vire et al. 2006). In this study, we reported that EZH2 mediated H3K27me3 at Clusterin promoter suppresses Clusterin expression via recruiting DNMT3a in GC-1spg cells, and knockdown study revealed that EZH2 served as a critical histone methylase in this process.

Clusterin was initially identified as a gene associated with sperm maturation in rat testis fluid (Wong et al. 1994). A recent study has reported that Clusterin is downregulated during DM-induced testicular damage. Reinforced Clusterin protects against HG-impaired cell viability and HG-induced cell apoptosis in GC-1spg cells via PI3K/AKT/mTOR signaling (Tian et al. 2021). In addition, an upstream regulatory axis of Clusterin has been demonstrated recently. METTL3-mediated m^6^A methylation stabilized TUG1 to enhance Clusterin mRNA stability in GC-1spg cells, thereby ameliorating DM-induced testicular damage (Tian et al. 2023). In accordance with the previous findings, we reported that Clusterin was suppressed in the testes from STZ mice and HG-treated GC-1spg cells. Downregulation of Clusterin was accompanied with the inactivation of AMPK and Nrf2 signalings, as well as the increased oxidative stress in HG-treated GC-1spg cells. AMPK activation rescues oxidative stress and inflammation in various diseases (Salminen et al. 2011; Lin et al. 2017). Our findings showed that Clusterin might regulate ferroptosis through AMPK signaling. AMPK and Nrf2 are closely related signalings (Petsouki et al. 2022), while how they work in concert to mediate Clusterin-modulated ferroptosis in the testis requires further investigation in the future study. It was worth noting that silencing of BRD7 reversed HG-downregulated GPX4/SLC7A11 in GC-1spg cells which was accompanied with the reduction of lipid ROS. These findings suggested that GPX4/SLC7A11 axis might also be implicated in BRD7-regulated ferroptosis, and the cross-talk between GPX4/SLC7A11 and AMPK signalings needs further investigation.

## Conclusion

In conclusion, we reported that BRD7 mediated Clusterin promoter hypermethylation via recruiting EZH2/DNMT3a, thereby suppressing AMPK signaling to facilitate ferroptosis and induce DM-associated testicular damage. These findings identified BRD7-associated ferroptosis as a promising therapeutic target for DM-induced male reproductive dysfunction. In the current study, the findings were validated in male mice and GC-1spg cells, and further clinical validation is required in the future study.

### Electronic supplementary material

Below is the link to the electronic supplementary material.

Supplementary Material 1

## Data Availability

The datasets generated during and/or analysed during the current study are available from the corresponding author on reasonable request.

## Abbreviations

AMPK: AMP-activated protein kinase
BRD7: Bromodomain protein 7
ChIP: Chromatin immunoprecipitation
co-IP: Co-immunoprecipitation
DM: Diabetes mellitus
DNMT: DNA methyltransferase
GC: Genome copies
GPX4: Glutathione peroxidase 4
H&E: Hematoxylin and Eosin
HG: High glucose
HSCs: Hepatic stellate cells
IF: Immunofluorescence
IHC: Immunohistochemistry
m^6^A: N6-methyladenosine
METTL3: Methyltransferase like-3
MSP: Methylation-specific PCR
Nrf2: Nuclear factor erythroid 2-related factor 2
OS: Osmotic stimulation
PBAF: Polybromo-associated BRG1-associated factor
PFA: Paraformaldehyde
PRC2: Polycomb repressive complex 2
PRMT5: Protein arginine methylatransferase 5
SLC7A11: Cystine/glutamate antiporter
STZ: Streptozotocin
