# Supplementary figures and images for: BRD7 facilitates ferroptosis via modulating clusterin promoter hypermethylation and suppressing AMPK signaling in diabetes-induced testicular damage
Source: Mol Med. 2024 Jul 12;30:100. doi: 10.1186/s10020-024-00868-x (PMC11241864; doi:10.1186/s10020-024-00868-x)

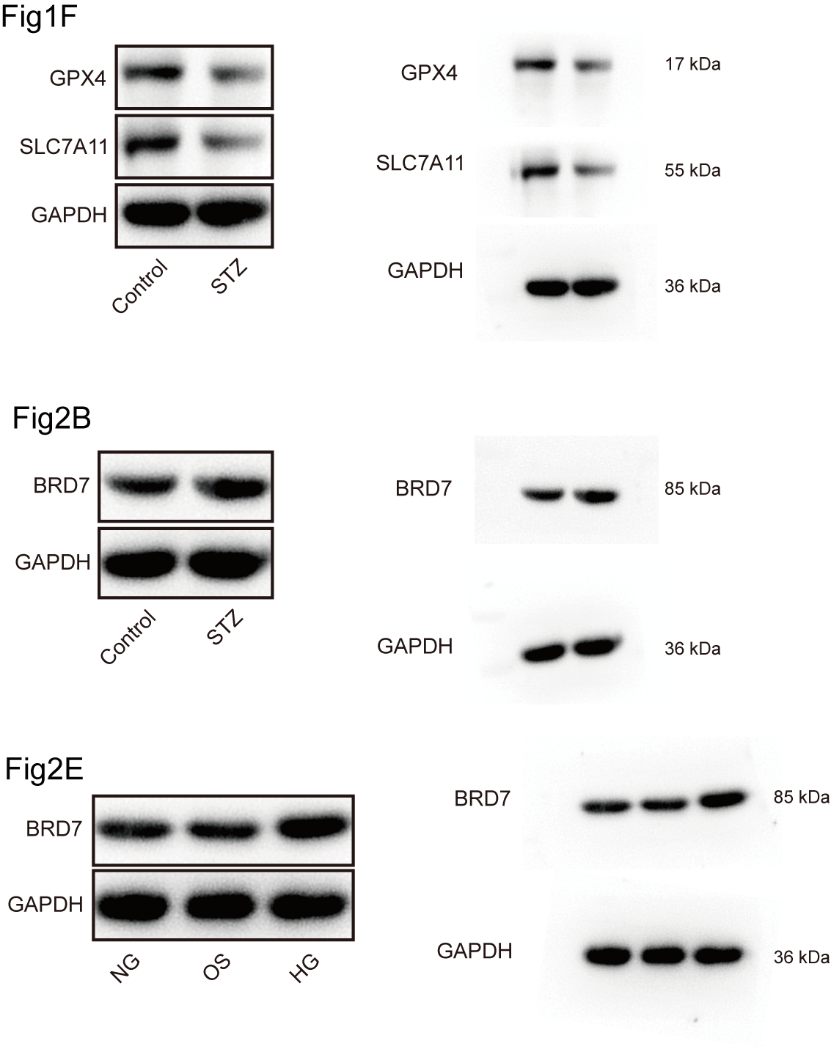


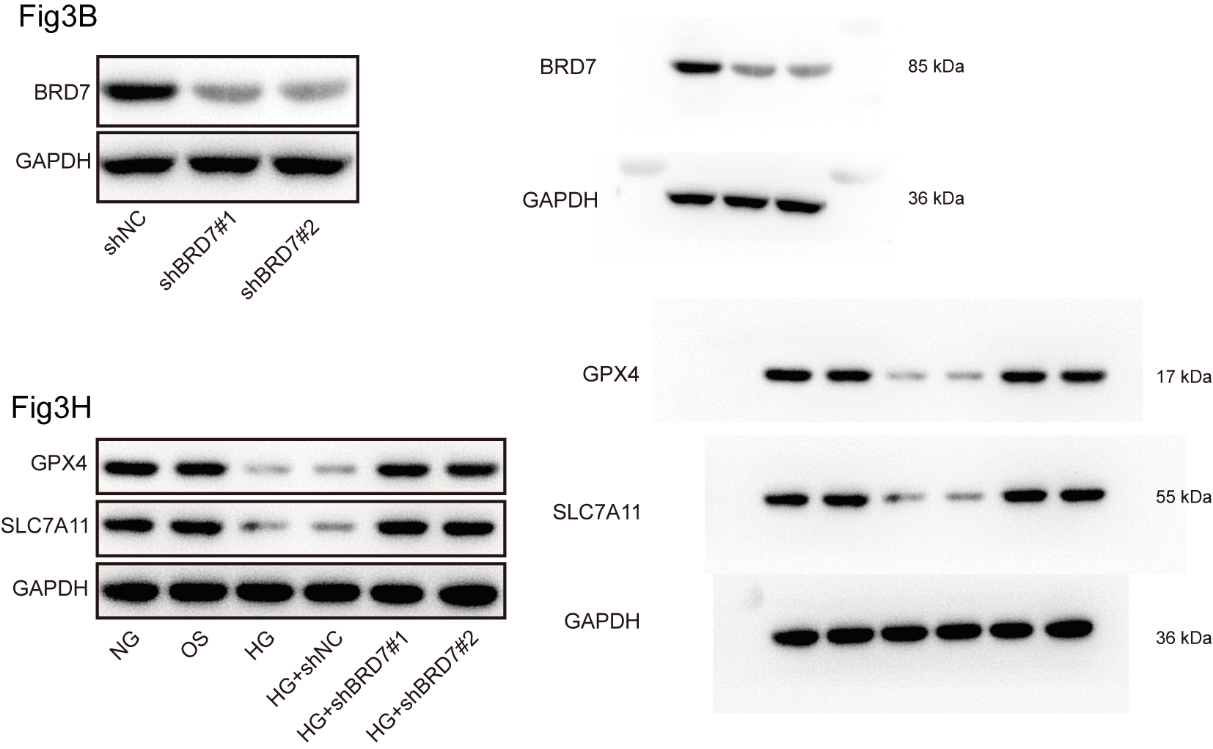


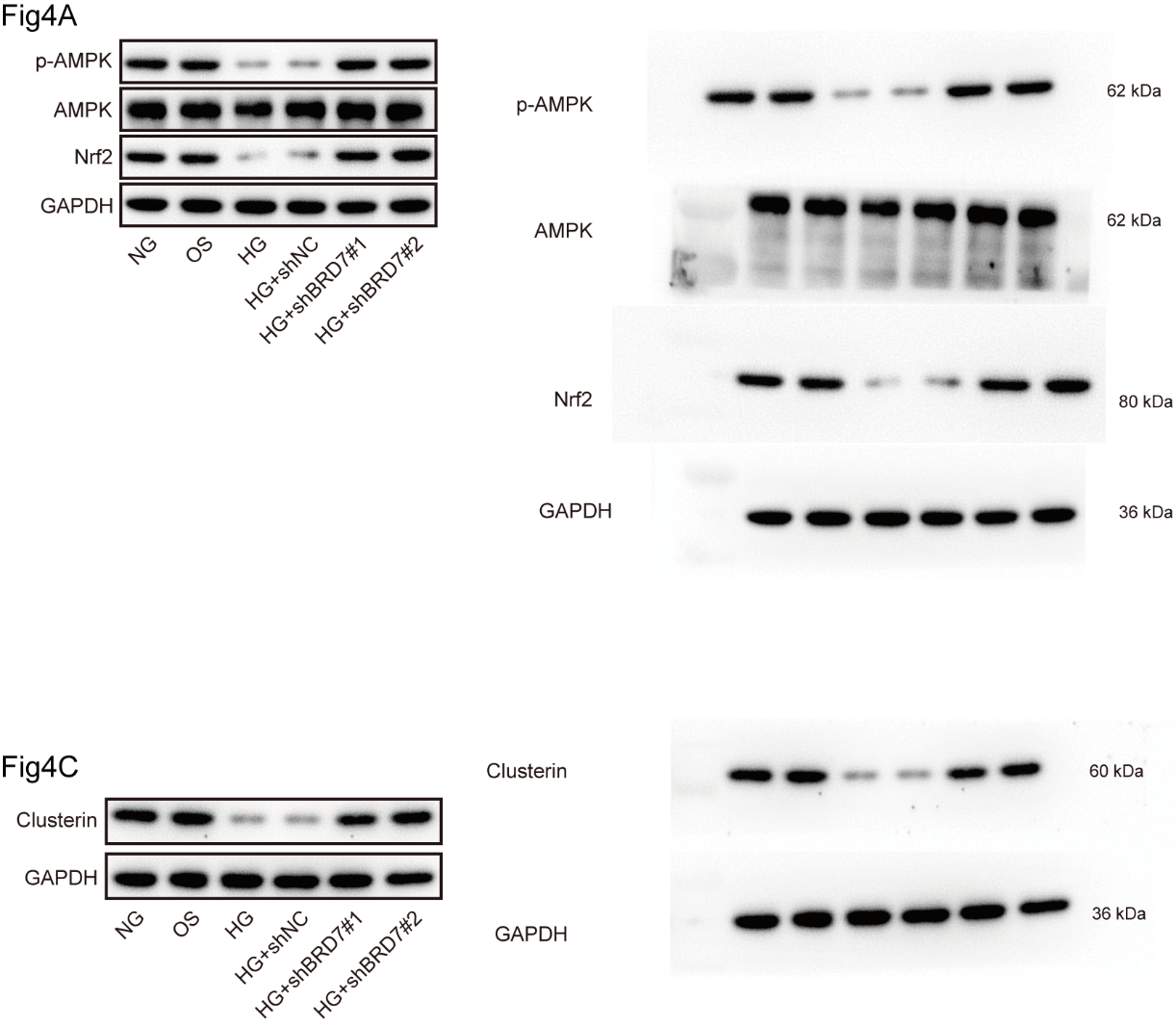


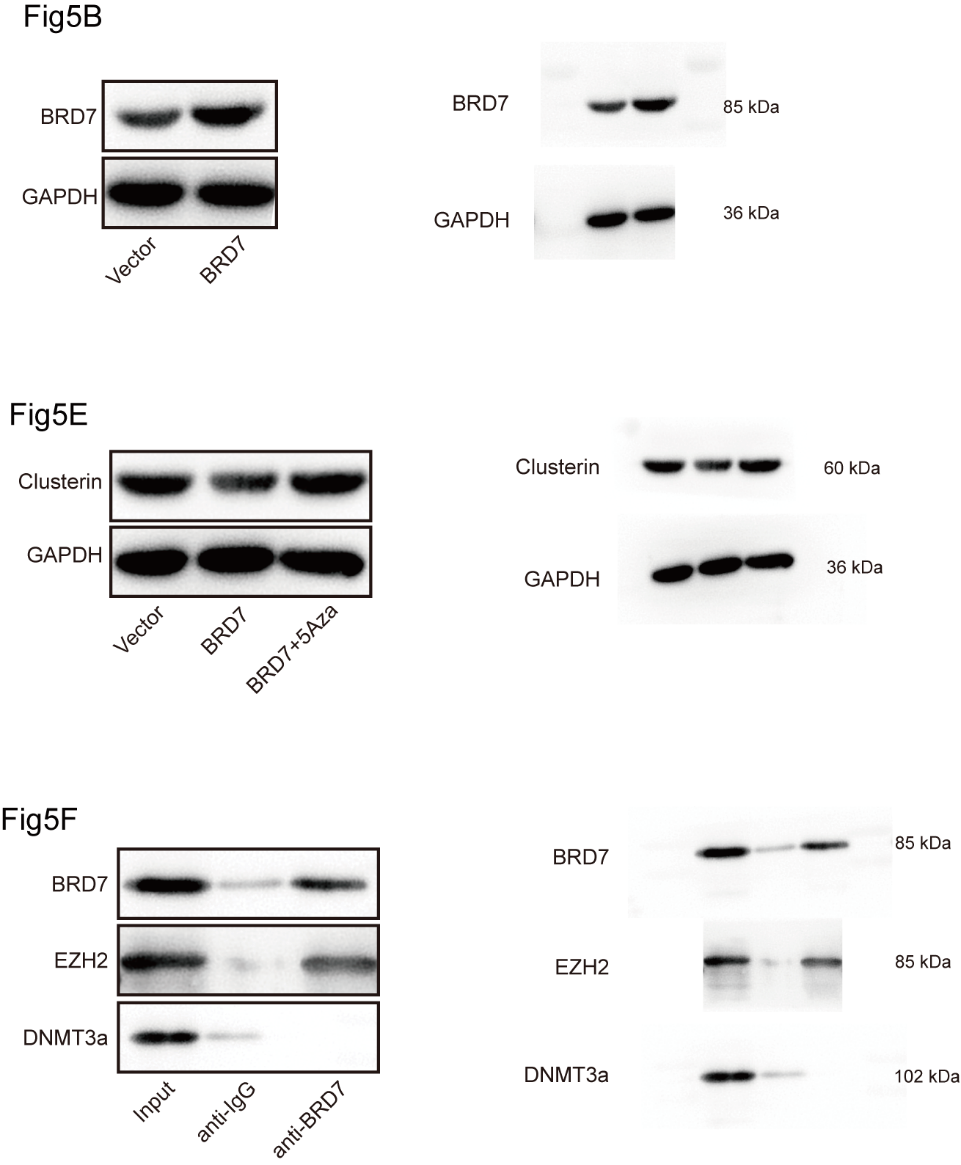


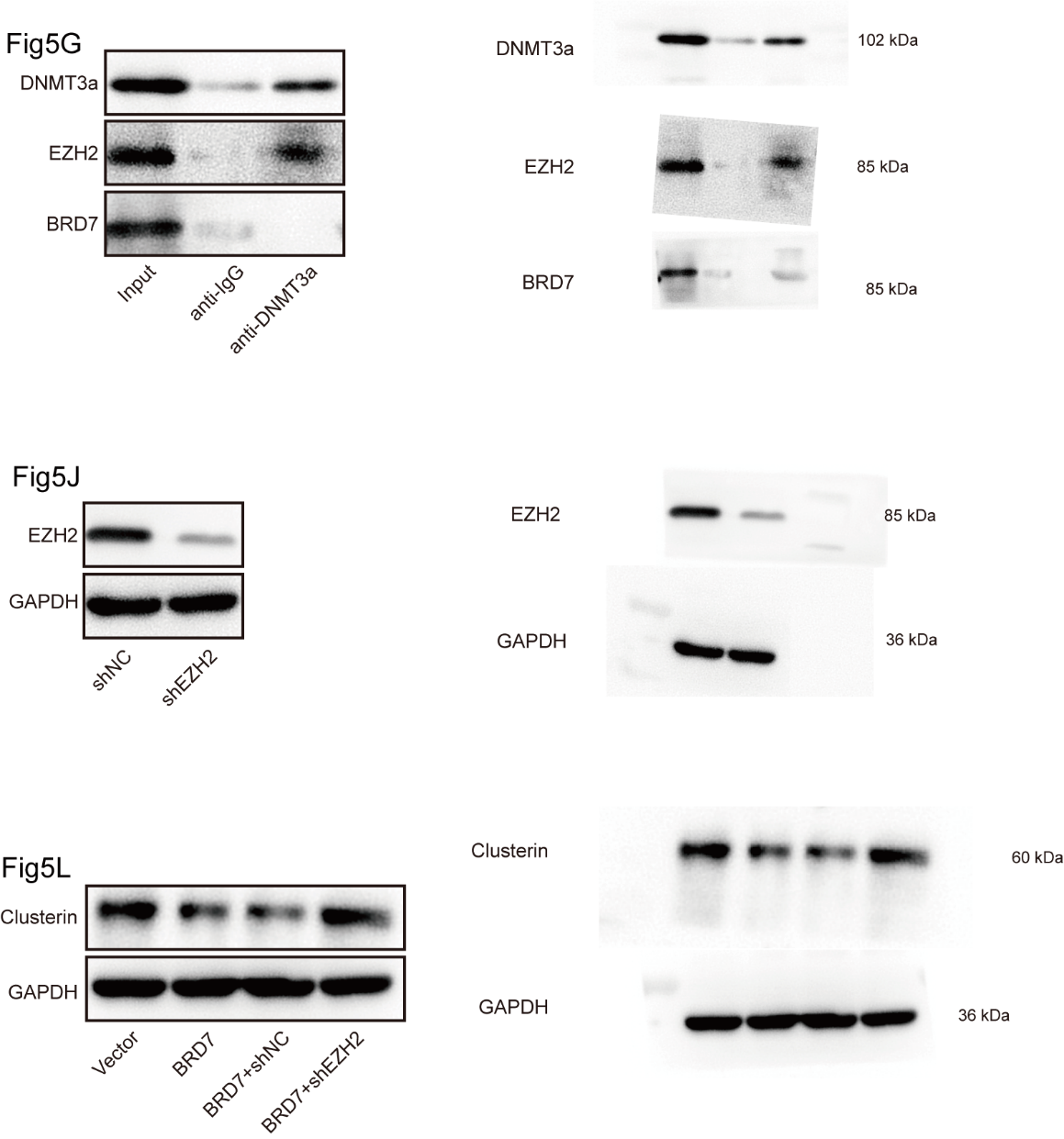


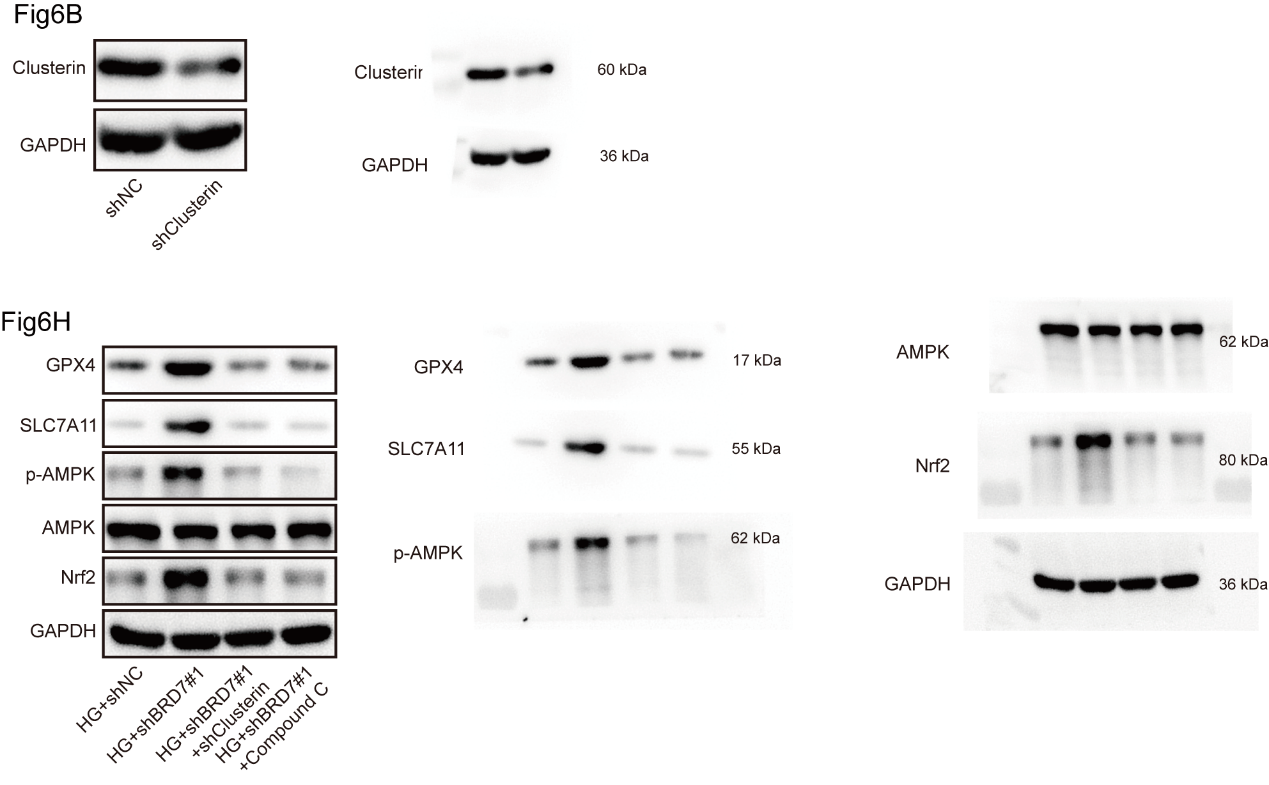


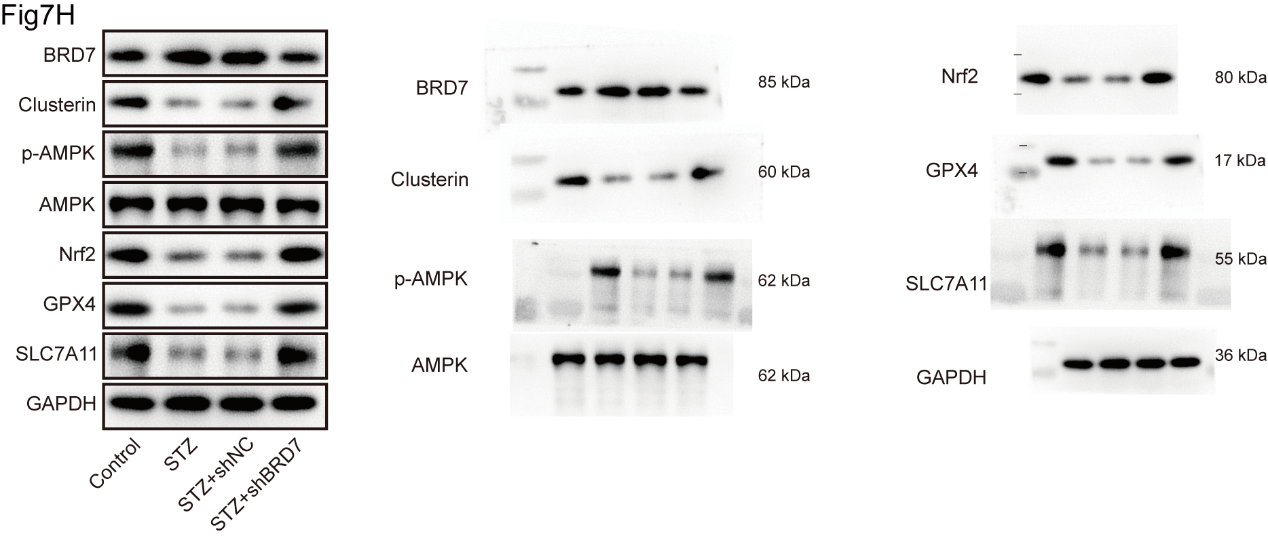

Supplement: Supplementary file 1 — Supplementary Material 1 [file 10020_2024_868_MOESM1_ESM.docx]
